# Fishing effort dynamics around the Galápagos Marine Reserve as depicted by AIS data

**DOI:** 10.1371/journal.pone.0282374

**Published:** 2024-04-03

**Authors:** Nicole Chinacalle-Martínez, Alex R. Hearn, Kristina Boerder, Juan Carlos Murillo Posada, Jean López-Macías, César R. Peñaherrera-Palma

**Affiliations:** 1 Pontificia Universidad Católica del Ecuador–Sede Manabí, Manabí, Ecuador; 2 MigraMar, Bodega Bay, California, United States of America; 3 Universidad San Francisco de Quito, Quito, Ecuador; 4 Dalhousie University, Halifax, Nova Scotia, Canada; 5 Centro Interdisciplinario de Ciencias Marinas, Avenida Instituto Politécnico Nacional, La Paz, Baja California Sur, México; Hellenic Center for Marine Research, GREECE

## Abstract

The waters around the Galápagos Marine Reserve (GMR) are important fishing grounds for authorized artisanal vessels fishing within the reserve as well as for national and foreign industrial fleets operating in the wider Ecuadorian Insular Exclusive Economic Zone (IEEZ). Although it was not originally designed for fisheries management, Automatic Identification System (AIS) data provides useful, open access, near real-time and high-resolution information that allows for increased monitoring, particularly around Marine Protected Areas (MPAs) and in Areas Beyond National Jurisdiction. This study uses AIS data provided by Global Fishing Watch to assess the spatial distribution and seasonal dynamics of fishing effort by vessel flag within the GMR and the IEEZ from 2012 to 2021. Based on kernel density estimation analysis, we determinate the core-use areas (50%) and spatial extent (95%) of fishing activities by fleets (Ecuadorian and foreign), gear types and seasons (warm, from December to May; and cold, from June to November). Our results show that the Ecuadorian fleet recorded the most observed fishing hours in the study area, with 32,829 hours in the IEEZ and 20,816 hours within the GMR. The foreign flags with the most observed fishing hours in the IEEZ were Panama (3,245 hours) and Nicaragua (2,468.5 hours), while in the GMR were the ‘Unknown flag’ (4,991.4 hours) and Panama (133.7 hours). Vessels fished employing different fishing gears, but the waters of the GMR and IEEZ were mostly targeted by tuna purse-seiners and drifting longlines. The spatial distribution of the fishing effort exhibits marked seasonal variability, likely influenced by seasonal migrations of target species such as tunas (e.g., *Thunnus albacares*, *T*. *obesus* and *Katsuwonus pelamis*), marlins (e.g., *Makaira nigricans*) and sharks (e.g., *Alopias pelagicus*). The collection and use of this type of spatial and seasonal information is an essential step to understand the dynamics of fishing activities in national waters and improve fisheries management, particularly in less studied areas and fisheries.

## Introduction

The delimitation of the Exclusive Economic Zones (EEZs) of coastal States (Third Conference of the United Nations Convention on the Law of the Sea, 1982) granted countries jurisdiction over the waters off their coasts and to control access to marine resources for their own benefit up to 200 nautical miles from the shore [1, 2]. However, many EEZs are still vulnerable to illegal, unreported, and unregulated (IUU) fishing due to the lack of resources for monitoring and controlling their waters [3]. Unsustainable fishing threatens the management of fishery resources, food security, and people’s livelihoods around the world [4] and is estimated to generate losses to countries’ tax revenues of up to four billion US dollars annually [5].

Remote vessel monitoring presents an opportunity to promote a new era of transparent marine governance and research [6]. The AIS is a system originally designed to prevent ship collisions [7], but due to the open access and continuous transmission of information of the identity, current position, speed and direction of a vessel, amongst other data, AIS data can also be used to obtain information on the activity and behavior of fishing vessels at a relatively low cost [8, 9]. The functioning of the AIS is similar to the Vessel Monitoring System (VMS), a methodology used by countries and regional management fisheries organizations to monitor commercial fishing [6, 10]; however, the latter is proprietary with high access barriers, generally transmits at lower intervals around every 30 minutes to two hours [8] and due to confidentiality issues shared VMS data is often aggregated at coarse resolutions [11]. Since AIS transmissions can be as frequent as every two seconds, its use represents an opportunity to extend monitoring and control in near real-time [8, 12], particularly around MPAs and even in remote Areas Beyond National Jurisdiction. However, the main shortcoming of AIS is that its use is only mandatory for vessels over 300 gross tons on international voyages as well as for some countries that have adopted its use (e.g. Europe Union), resulting in uneven spatiotemporal coverage, particularly in the early years of the systems’ implementation [13].

The GMR is an MPA of approximately 138,000 km^2^ around the Galápagos Islands created in 1998 by the Government of Ecuador [14]. It is a key area for the conservation of numerous threatened marine species, including 13 species of seabirds [15], 576 bony fishes, 56 species of sharks and rays [16], six species of marine mammals [17], six species of marine reptiles [18], and 25 endemic species to the Galápagos Islands [19]. Since its creation, industrial fishing with any type of hydraulic, mechanized and technical fishing gear within the MPA has been prohibited [20]. The creation of GMR led to an increase in fishing productivity, but also to a change in the behavior of vessels that increased their fishing effort around the borders of the marine reserve (known as "fishing the line”) [21, 22]. Artisanal fishing within in the GMR is allowed from vessels less than 18 meter in length and 50 gross tons, which may only use manual fishing gear described in the Galápagos Fishing Regulation (e.g., trolling, deep sea and mid-water hook and lines, coastal gillnets, and Hawaiian slings) [23]. The fishing activities in GMR are controlled by the Directorate of the Galápagos National Park and the Ecuadorian National Navy [23]. The technology used for marine control and surveillance in the GMR includes a radar system, the VMS and recently the AIS [24]. Since 2014, the use of AIS has been mandatory for all tourist and artisanal fishing vessels under 20 gross tons operating in the GMR [24].

The GMR is surrounded by a maritime area of Ecuadorian jurisdiction that extends 200 nautical miles from the Galápagos Islands baseline (IEEZ) [25]. Important activities such as industrial fishing and tourism, key components of the Ecuadorian economy, are actively developed in this area and constitute 0.7% of the Ecuadorian Gross Domestic Product [26, 27]. Industrial fishery catches in the IEEZ are comprised of 89% tunas (*T*. *albacares*, *T*. *obesus*, *K*. *pelamis*), as well as Humboldt squid (*Dosidicus gigas*) and blue shark (*Prionace glauca*) [28]. Compared to the GMR, the planning and management of the IEEZ is inter-institutional and includes different governmental levels [29]. The spatio-temporal dynamics of the purse seine and longline tuna fishery active in the IEEZ has been previously studied using catch and effort data from on-board observer programs [21, 30] providing information on current status, effort distribution and catch composition from the Regional Fisheries Management Organization in charge (Inter-American Tropical Tuna Commission, IATTC) [31]. However, the fishing effort allocation of other gear types and from other nations without on-board observer programs is unknown and, when available, is often aggregated at coarse spatial resolution (e.g., 1° or 5° grid cells) not suitable for the analysis of fishing patterns [32].

Since 2012, non-governmental organizations such as Global Fishing Watch have been generating high-resolution vessel activity data (e.g. 0.01° grid cells) using AIS to promote transparent and sustainable fisheries [33]. The potential of this tool to advise GMR managers and authorities has been previously assessed to characterize fishing effort around IEEZ [34] and the interactions of the tuna purse-seine fishing fleets with the GMR [22]. This type of information can be particularly useful for institutions or stakeholders in charge of management and law enforcement that do not have access to it or do not have the necessary tools to process it.

This study provides a detailed evaluation of the spatial as well as seasonal patterns of fishing activity by different flag states in the IEEZ and GMR. Particularly, this study aims to 1) describe the total fishing hours per flag state and gear type of each fleet; 2) determine the fishing location distance from the IEEZ; and 3) explore the spatial and seasonal variation of the fishing effort using AIS data obtained from Global Fishing Watch.

## Materials and methods

### Study area

**Fig 1:**
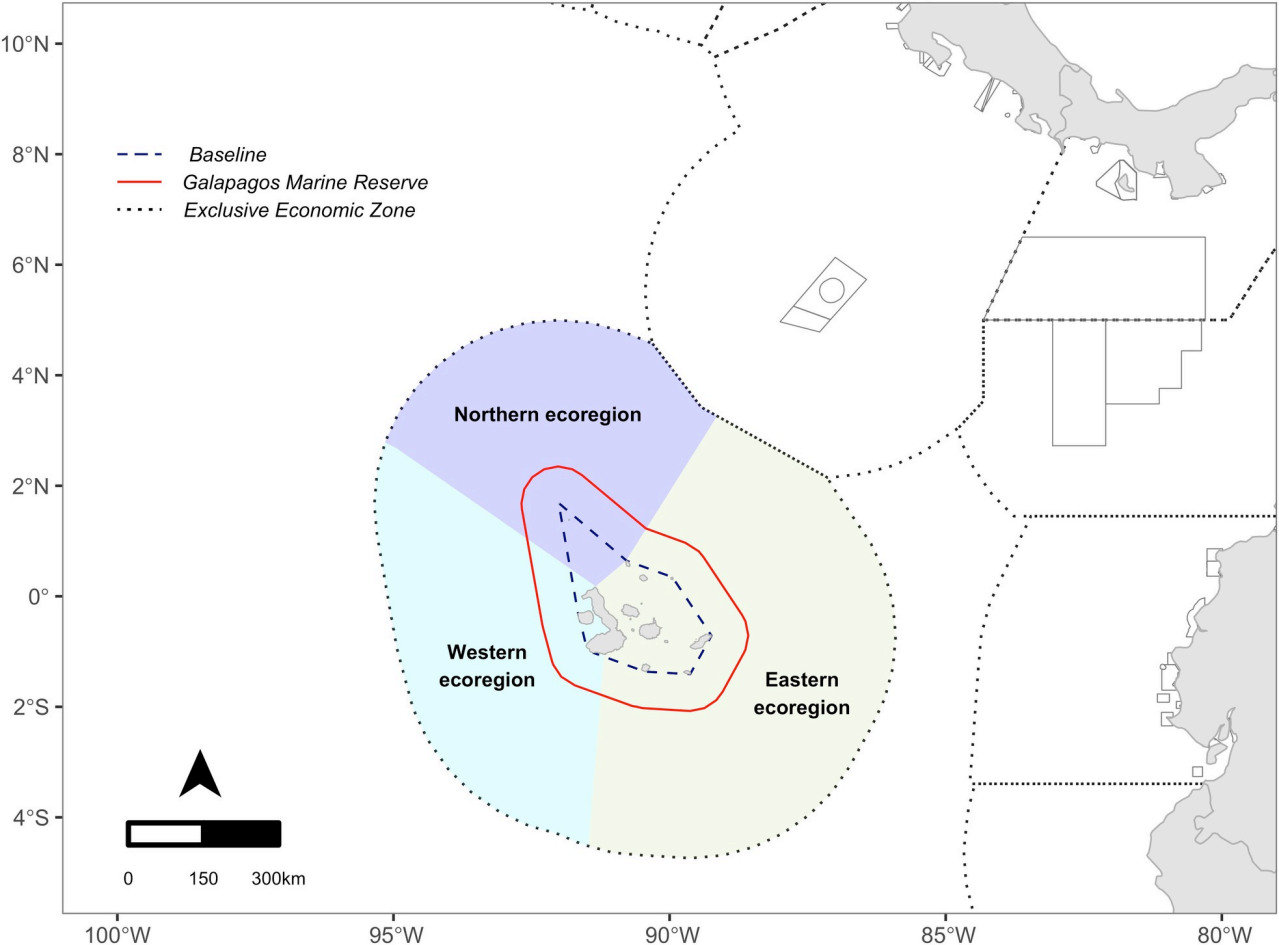
Study area location. Ecoregions on the map are based on Spalding, Fox [38]. The GMR includes the entire marine area within a 40 nautical mile (76 km) strip, measured from the archipelago baseline (dashed line) [39]. The MPA shown in the map are those created until 2021.

The Galápagos Islands lie in the Eastern Tropical Pacific Ocean, approximately 970 km away from the continental coast of Ecuador. The archipelago comprises 19 major islands and more than 200 islets and rocks [35]. The oceanographic conditions around the Galápagos can be divided into two seasons: a cold season with temperatures between 18–20°C (from June to November), and a warm season with temperatures > 25°C (from December to May) [36, 37]. To evaluate the spatial distribution of fishing effort by zone, we used the marine ecoregions described by Spalding, Fox [38]: The Northern Galápagos Islands Ecoregion (northern ecoregion), the Eastern Galápagos Islands Ecoregion (eastern ecoregion) and the Western Galápagos Islands Ecoregion (western ecoregion) (Fig 1 and Table 1).

**Table 1:** Characteristics of Galápagos Islands Ecoregions based on Spalding, Fox [38]. Oceanographic characteristics taken from: Dirección del Parque Nacional Galápagos [40], Liu, Xie [37] and Sullivan and Bustamante [41]. Area estimated in km^2^ using R Core Team [42].

Ecoregion	Area (km^2^)	Islands	Oceanographic characteristics
Northern ecoregion	213,063.5	Darwin and Wolf.	Influenced by warm waters of the Panama Current that reach temperatures around 28°C.
Eastern ecoregion	390,699.1	Pinta, Marchena, Genovesa, Santiago, Pinzón, Baltra, Santa Cruz, Santa Fe, San Cristóbal, Floreana, Española and the eastern part of Isabela.	Influenced by the cold waters of the Humboldt Current and has temperatures of approximately 24°C.
Western ecoregion	232,030.3	Fernandina and the western part of Isabela.	The convergence of the Cromwell current with the Galápagos shelf creates upwelling conditions associated with high primary productivity and temperatures around 14°C.

### Data extraction

We downloaded daily fishing effort information from the Global Fishing Watch database version v20201001 [43]. Global Fishing Watch analyzes more than 50 million AIS messages daily and identifies fishing vessels and their fishing activities using two machine learning algorithms for image classification called convolutional neural network (for more information see supplemental materials of Kroodsma, Mayorga [44]). The first model analyzes all vessels AIS positions and predicts the vessel type (fishing or non-fishing vessels) incorporating a set of official registries of vessels with information on vessel identity and characteristics (length, engine power, tonnage). The current model is relatively accurate to identify fishing vessels from non-fishing vessels (99%) [44]. For fishing vessels, gear type is assigned when the information in the official registry matches the classification of the model [12]. Among the fishing gears identified by the model are drifting longlines (LONG), pole and line (POLE), set gillnets (GILL), set longlines (SETL), squid jiggers (SQUID), other purse-seines (PURS), tuna purse-seines (TUNA) and unknown (UNKN). Set gillnets and set longlines corresponds to gear fixed to the seabed [45]. “Unknown” gear is a generic category assigned to those fishing vessels for which the type of fishing gear used cannot be clearly determined.

The second model identifies the apparent activity of a vessel in a specific location based on the speed of the vessel, the change of direction within a defined area, spatio-temporal patterns of movement, and duration of the fishing event. The accuracy scores vary depending on the fishing gear, but in general range above 90% [44]. To convert the apparent fishing activities into fishing hours, an amount of time is assigned to each AIS detection (which is assigned half the time to the previous and next AIS position) and all positions in each grid are summed [12]. The final product of this model identifies the location and duration of fishing activity, which can be aggregated per grid cell (hereafter “hours”), flag state and gear type and summarized by day. The data used in this study was aggregated on a grid with 0.01 decimal degrees (approx. 1.23 km^2^) resolution [43].

For this investigation, we filtered the data downloaded from Global Fishing Watch to obtain only information of fishing vessels within the IEEZ and GMR areas from January 2012 to December 2021. To avoid confusion with the legal artisanal fisheries that can operate in Galápagos, we filtered the Ecuadorian-flagged vessels and used information from Ecuador’s National Public Registry of Vessels to identify which vessels were industrial and artisanal [46]. For those vessels that were not in the public registry, we reviewed their range of movement within the Global Fishing Watch iterative map [47]. Those vessels that limited their movements within the GMR were categorized as artisanal vessels. Finally, we categorized data with missing flag information as ‘Unknown flag’ regardless most of its fishing effort was allocated within the GMR (S1 Fig). Since the lack of a Maritime Mobile Service Identity (MMSI) number prevented assigning it to any of the local, national or foreign fleets, we only used this data for comparative purposes within the descriptive statistical section and eliminated it from further analysis.

### Data analysis

Due to the annual increase of the number of fishing vessels transmitting AIS and the number of satellites receiving the signals [12] temporal coverage of the Global Fishing Watch data varies over the years, we aggregated date over the years to reduce the bias. The dist2Line function of the ‘geosphere’ package [48], available in the R computer software version 2022.02.0 [42], was used to determine the boats’ entry distance into the study area. This function calculates the shortest perpendicular distance between points and polygons and generates a matrix with the distance and longitude/latitude of the point closest to the polygon (S2 Fig) [48]. We calculated the entry distance to the IEEZ from each AIS detections to the edge of the IEEZ polygon, while we calculated the entry distance to the GMR from AIS detections to the edge of the GMR polygon. Boxplots were used to compare the foreign and Ecuadorian vessels’ entry distance per flag state, gear type, seasons and ecoregions.

A utilization distribution model using Kernel Density Estimators (Kernel UD) was used to assess the core effort areas and spatial extent of fishing activities by vessel type (foreign and national), fishing gear and seasons. Kernel UD analysis defines the minimum area in which a point has the specific probability of being located according to its coordinates [49]. We used the kernelUD function available in the ‘adehabitatHR’ package of the R software version 2022.02.0 [42, 50], using the reference bandwidth, the extent of the IEEZ (longitude: - 85.90 to—95.34 and latitude: - 4.73 to 4.99) and a grid size constructed with a resolution of 0.01 degrees. We used 50% Kernel UD to estimate core-use areas and 95% Kernel UD to estimate the spatial extent of fishing. Two graphs were made for each variable analyzed: one integrating fishing data from all the known foreign fishing fleets, and another just for the Ecuadorian fleet.

## Results

According to Global Fishing Watch algorithms, a total of 49,607 AIS detections by 147 unique vessels (based on the MMSI) were recorded within our study area from 2012 to 2021. In the IEEZ, 36,295 AIS detections belonging to 111 vessels from 13 flag nations, including Ecuador, were recorded; while in the GMR, 13,312 AIS detections belonging to 49 vessels from nine of those nations (including Ecuador and ‘Unknown flags’), were recorded (S1 Fig). Ecuadorian industrial vessels were detected mainly around the IEEZ, while artisanal vessels within the GMR. Foreign vessels were detected mostly in the northern and western IEEZ, with some (i.e., Japan, Panama, Vanuatu and Nicaragua) detected also within the GMR. ‘Unknown flag’ was the only category to report more detections within the GMR (> 2,400) than in the IEEZ (< 120) (Table 2).

**Table 2:** Summary statistics for fishing effort per vessel flag as seen from AIS data. Abbreviations: N, number of vessels; Cells, number of grid cells (0.01 decimal degrees) associated with fishing activity; Sum, the quantity of fishing hours per category; Mean, the arithmetic mean; sd, the standard deviation. Only the flag state category contains information on ‘Unknown flags’ (UNK).

Category	Total fishing hours					Fishing hours only in IEEZ					Fishing hours within GMR				
	N	Cells	Sum	Mean	sd	N	Cells	Sum	Mean	sd	N	Cells	Sum	Mean	sd
**Flag state**															
China (CHN)	4	4	16.2	4	4.1	4	4	16.2	4	4.1	0	0	-	-	-
Colombia (COL)	10	1,144	1,687.8	1.4	1.4	10	1,142	1,683.8	1.4	1.4	1	2	4.0	1.3	0.3
Ecuador (ECU)	86	29,302	53,945.2	1.3	1.9	60	26,053	33,128.5	1	1.0	32	3,249	20,816.6	1.9	3.1
Japan (JPN)	1	2	1.9	0.9	0.5	0	0	-	-	-	1	2	1.9	0.9	0.5
Nicaragua (NIC)	1	2,491	2,488.0	0.9	0.4	1	2,473	2,468.5	0.9	0.4	1	18	19.4	1.0	0.4
Panama (PAN)	10	2,304	3,378.7	1.4	1.5	9	2,226	3,245.0	1.4	1.5	2	78	133.7	1.6	1.4
Portugal (PRT)	1	1	2.4	2.4	0	1	1	2.4	2.4	0.0	0	0	-	-	-
Spain (ESP)	7	49	42.8	0.8	0.3	7	49	42.8	0.8	0.3	0	0	-	-	-
Taiwan (TWN)	1	15	25.3	1.6	1.4	1	15	25.3	1.6	1.4	0	0	-	-	-
United Kingdom (GBR)	1	29	23.7	0.8	0.5	1	29	23.7	0.8	0.5	0	0	-	-	-
United States (USA)	4	159	179.9	1.1	1.1	4	140	142	1	0.6	2	19	37.8	1.6	2.6
Unknown (UNK)	10	1,065	5,115.4	1.9	3.4	3	327	123.9	1	0.5	7	738	4,991.4	2.0	3.5
Vanuatu (VUT)	1	2	0.8	0.4	0.5	0	0	-	-	-	1	2	0.8	0.4	0.5
Venezuela (VEN)	10	2,029	2,107.9	1	0.6	10	2,024	2,104.7	1	0.6	2	5	3.1	0.6	0.3
**Gear type**															
Tuna purse-seines (TUNA)	84	24,756	28,760.4	1.0	0.9	84	24,714	28,714.4	1.0	0.9	9	42	45.9	1	0.7
Unknown gear types (UNKN)	16	1,988	12,515.5	1.8	3.0	2	7	18.4	2.3	2.7	15	1,981	12,497.1	1.8	3.0
Drifting longlines (LONG)	18	8,875	14,109.7	1.4	1.3	16	8,871	14,106.9	1.4	1.3	2	4	2.7	0.6	0.5
Set longlines (SETL)	8	776	4,902.8	1.9	3.2	0	0	-	-	-	8	776	4,902.8	1.9	3.2
Pole and line (POLE)	5	672	2,881.1	2.2	3.2	2	20	22.6	1.1	0.6	5	652	2,858.5	2.2	3.3
Set gillnets (GILL)	1	145	392.6	2.2	2.9	1	1	5.1	5.1	0.0	1	144	387.4	2.2	2.9
Other purse-seines (PURS)	2	89	323.0	1.9	3.1	0	0	-	-	-	2	89	323.0	1.9	3.1
Squid jigger (SQUID)	3	2	15.7	5.2	4.1	3	2	15.7	5.2	4.1	0	0	-	-	-
**Ecoregion**															
Eastern (EE)	123	10,131	24,815.2	1.5	2.3	93	7,599	8,522	1.0	0.9	37	2,532	16,355.1	1.8	2.9
Northern (NE)	78	12,433	18,304.8	1.3	1.2	71	12,192	17,595.3	1.3	1.1	9	241	713.3	1.7	2.6
Western (WE)	93	13,689	17,508.5	1.1	1.2	76	13,448	16,536.7	1	1.0	25	241	1,205.0	2.1	3.1
**Season**															
Cold	123	21,966	36,798.1	1.3	1.8	94	20,013	25,590.9	1.2	1.1	36	1,953	11,207.1	1.9	3.1
Warm	114	15,763	26,804.0	1.3	1.7	86	13,954	16,993.4	1.1	0.9	36	1,809	9,810.60	1.9	2.9

In the IEEZ from 2012 to 2021, the Nicaragua flag vessels recorded the most fishing activity overall, with 2,468.50 hours carried out by only one vessel (Table 2). Ecuador was the second nation that fished the most in this area (627.2 hours per vessel), followed by Panama (337.8 hours per vessel) and Venezuela (210.7 hours per vessel). In the case of GMR, the ‘Unknown flag’ vessels recorded the most fishing activity (713 hours per vessel), followed by the Ecuador (650.5 hours per vessel) and Panama (66.8 hours). China reported the highest mean fishing hour (4 hours) of all countries; however, this was carried out by only four vessels that fished for a total of 16.2 hours. In a similar scenario, Portugal was second in mean fishing hours (2.4 hours) and did it one time with one single vessel. Vanuatu reported the lowest mean fishing hour (0.4 hours) and the lowest total fishing hours (0.8 hours), also carried out by one boat.

**Fig 2:**
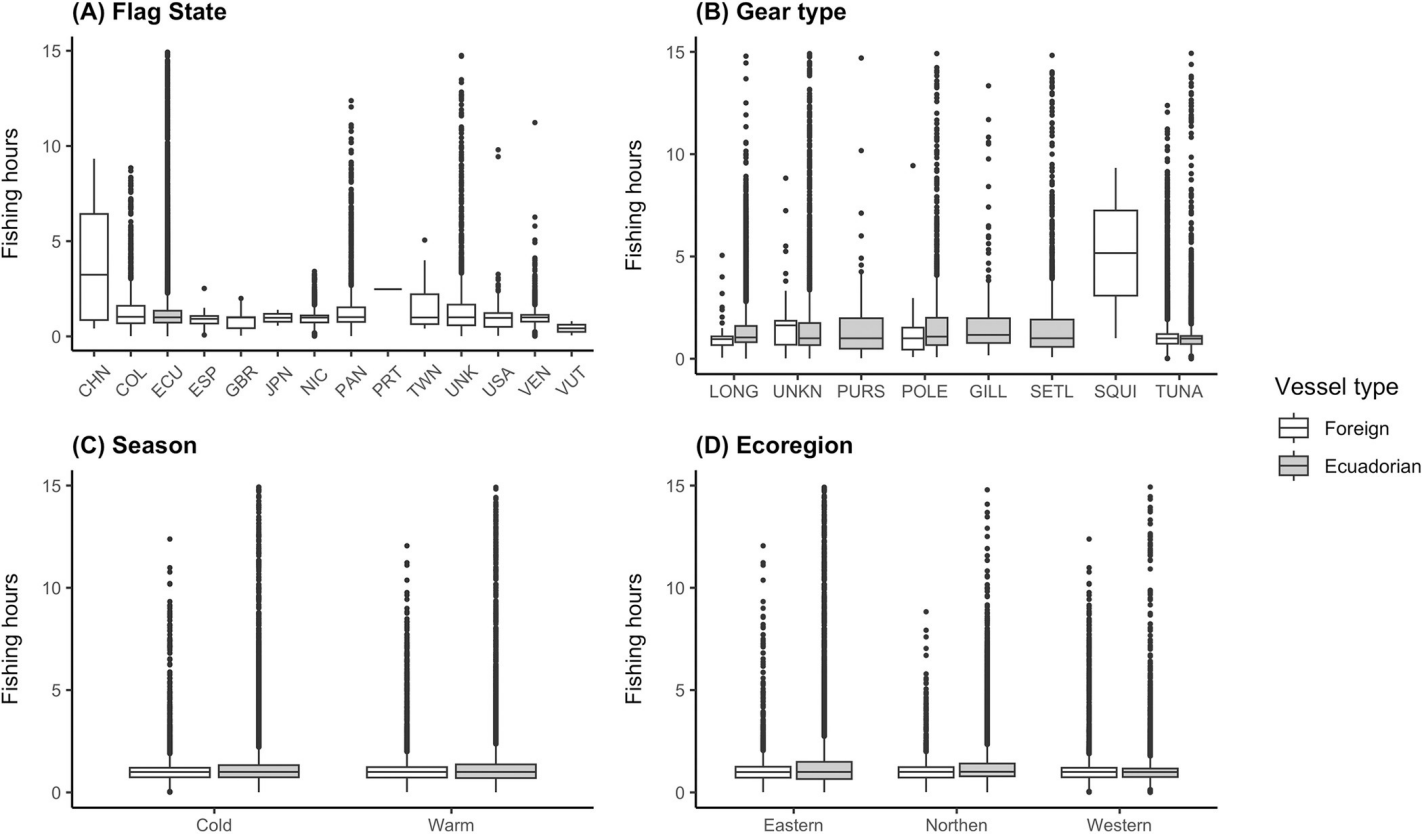
Fishing effort distribution in the IEEZ and the GMR per (A) flag state, (B) gear type, (C) season, and (D) ecoregion. Data from ‘Unknown flags’ were only used in flag state plot. The y-axis was standardized to 15 hours for better visual representation of the data. The outliers for ECU, PAN, UNK, POLE, SETL, TUNA, Cold, Warm, Eastern and Western are over 25 hours.

Vessels fished in the IEEZ and the GMR employing a variety of fishing gear, from tuna purse-seines (84 vessels), drifting longlines (18 vessels), pole and line (five vessels), set longlines (eight vessels), squid jiggers (three vessels), set gillnets (one vessel), and other purse-seines (two vessels) (Fig 2B). A total of 16 vessels predicted to be engaged in fishing activities were associated with unknown gear types. Squid jiggers were recorded to operate with the highest mean (5.2 hours) and were only used by foreign fleets. In the IEEZ, vessels fishing with tuna purse-seines and drifting longlines accumulated the highest amount of fishing hours (28,415.4 and 14,106.9 hours, respectively), while in the GMR, unknown gear types and set longlines accumulated the most (12,497.1 and 4,902.8 hours, respectively). The total amount of fishing hours was higher in the cold season for both the IEEZ and GMR, yet there was no difference when considering the mean fishing hours for only the GMR and the total dataset (IEEZ and GMR combined). Within the IEEZ, the cold season exhibited a higher mean fishing hour (1.2 hours; Fig 2C). The eastern ecoregion exhibited the highest mean (1.5 hours) and total (24,815.2 hours) fishing hours, yet it was the lowest when referring only to fishing hours within the IEEZ (Fig 2D). The northern ecoregion held the highest mean fishing hours (1.3 hours) in relation to the IEEZ, while the western held the highest mean fishing hours (2.1 hours) when considering only the data from the GMR.

**Fig 3:**
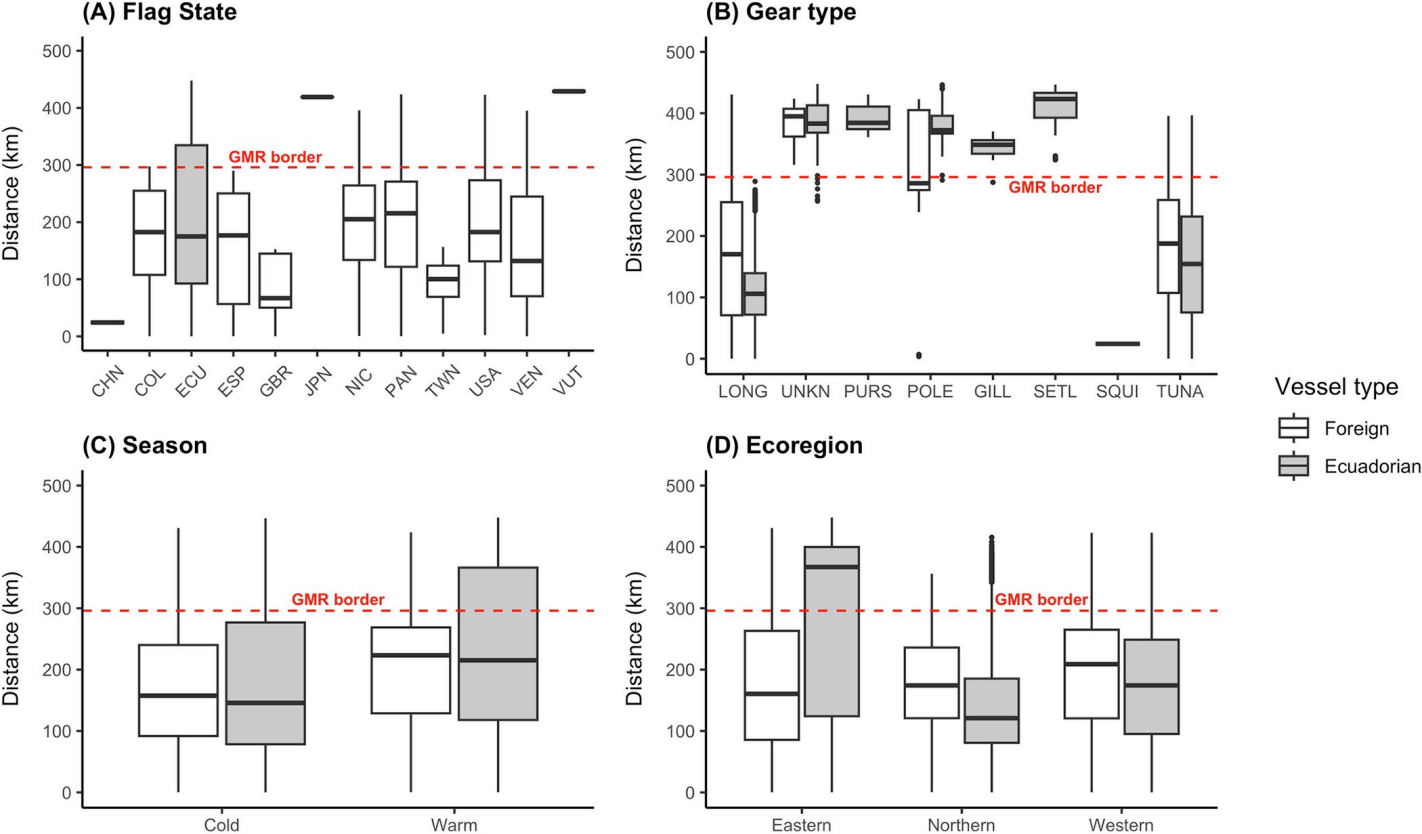
Entry distance to the IEEZ and the GMR per (A) flag state, (B) gear type, (C) season, and (D) ecoregion. The segmented red line represents the boundary between the IEEZ and the GMR (0 to 296 km corresponds to IEEZ; 296 to 500 km corresponds to the edge of the GMR towards its interior). Data from ‘Unknown flags’ were not used in these plots.

Vessels fished at a mean distance of 202 km from the edge of the IEEZ. China and Portugal operated closer to the IEEZ outer limit (12.7 km and 2.1 km, respectively), while the vessels from Japan and Vanuatu registered fishing events only inside the GMR (419 km and 429 km, respectively) (Fig 3A). These two were the farthest incursions in the GMR registered within this study. Other countries fished over larger areas across the IEEZ and GMR. For example, Ecuador, Panama, and Nicaragua fished with a mean distance of 207.2 km, 193.6 km, and 194 km, yet records expanded from the edge of the IEEZ towards well within the GMR.

All fishing gears excepting squid jiggers were detected within the GMR (Fig 3B). Set longlines exhibited the highest entry distance within the GMR, at a mean entry distance of 414 km (median of 423 km). In the warm season, foreign vessels operated closer to the GMR limit at a mean distance of 196.9 km (median of 222.9 km) and Ecuadorian vessels at 230.9 km (median of 225 km; Fig 3C). During the cold season, foreign vessels operated farther from the GMR border at a mean distance of 158.8 km (median of 156 km) and Ecuadorian vessels at 191 km (median of 152 km). Incursions into the GMR occurred in all ecoregions and seasons. In the northern and western ecoregions, fishing by foreign and national flags occurred mainly in the IEEZ at a mean entry distance of 139 km and 178 km, respectively (median of 124 km and 184 km, respectively; Fig 3D). Differently, fishing in the eastern ecoregion by Ecuadorian vessels occurred mostly in the GMR at a mean distance of 279 km (median of 367 km), while the foreign vessels did it mostly in the IEEZ at a mean distance of 168 km (median of 160 km).

**Fig 4:**
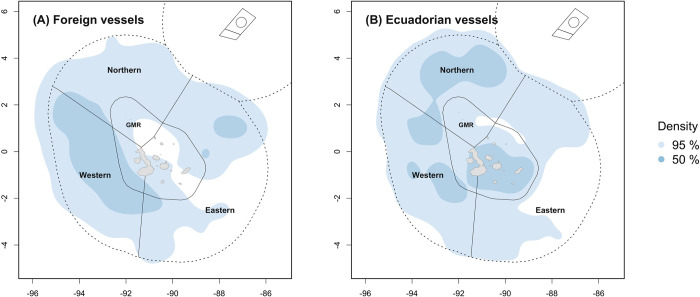
Spatial distribution of fishing effort during 2012–2021. (A) Kernel density map with fishing data for all foreign vessels. (B) Kernel density map only with fishing data of the Ecuadorian vessels. Data from ‘Unknown flags’ were not used in these plots.

**Fig 5:**
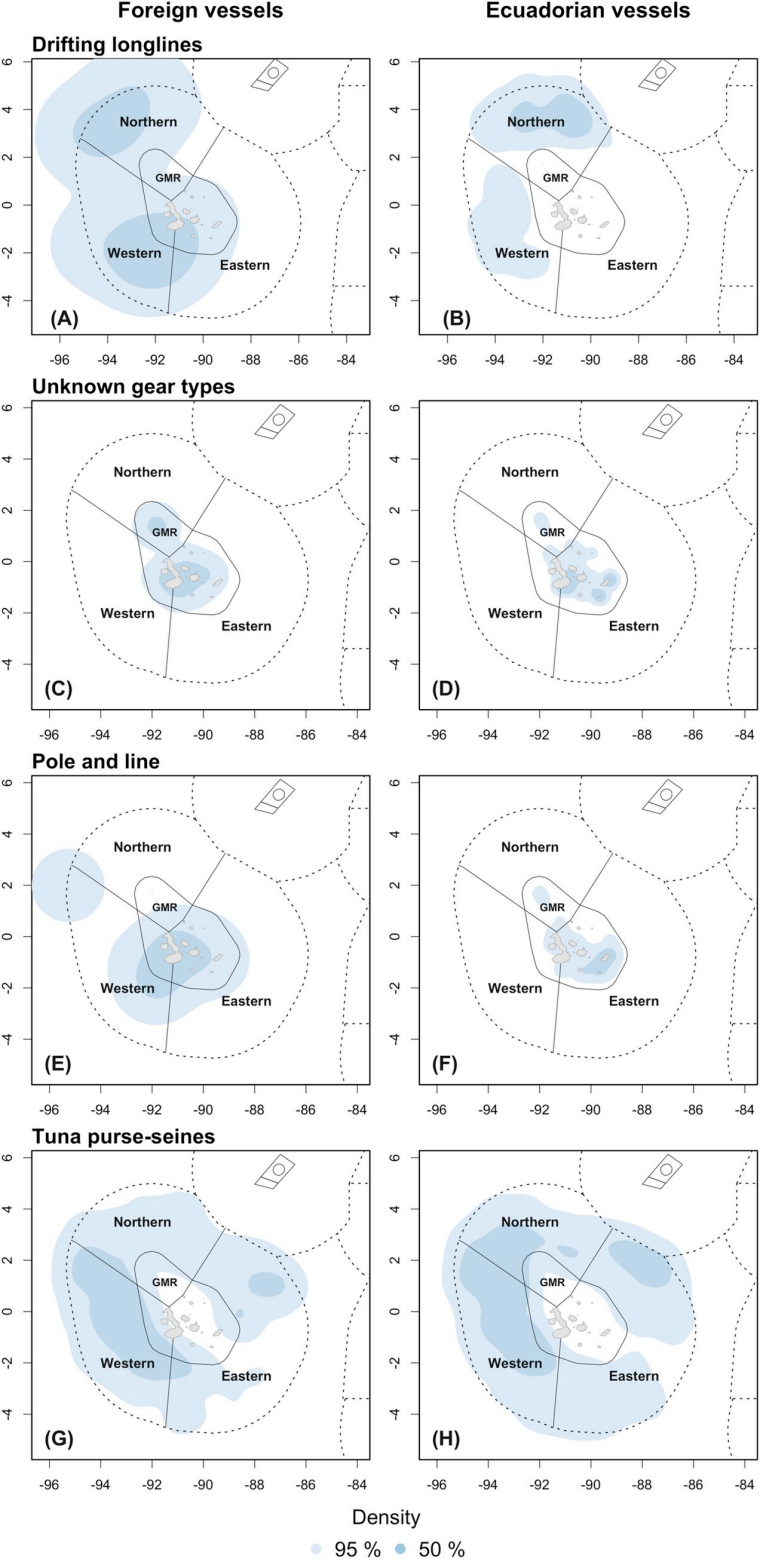
Spatial distribution of fishing effort by fishing gear. Fishing gears displayed here are those used by both fleets and that recorded enough information during the study period. Data from ‘Unknown flags’ was not used in these plots.

For the entire study period, fishing activity occurred the most in the northern and western ecoregions of both the IEEZ and GMR (Fig 4 and Table 3). Regarding the foreign fleets, the estimated core fishing effort area was located mostly in the western IEEZ, covering nearly 53% of that ecoregion and along the limit of the GMR (Fig 4A). For the Ecuadorean fleet, core areas were estimated to cover 39.6% and 22.4% of the IEEZ at the northern and western ecoregions, respectively, whilst within the GMR, the core area covered 57.7% of the eastern ecoregion (Fig 4B). Fishing effort by gear type also showed an important core area in the western ecoregion for the foreign fleets, excepting for the unknown gears and pole and line which were mostly allocated within the GMR (Fig 5 and Table 4). Either for the foreign or Ecuadorian fleet, tuna purse-seines allocated their fishing effort throughout the entire IEEZ, covering from 61 to 93% of the area at each ecoregion. Yet, the core area of the foreign fleets was more concentrated towards the western ecoregion of both the IEEZ and the GMR.

**Table 3:** Percent of overlap of core effort areas (50% kernels) and spatial extent of fishing activities (95% kernels) by fleets.

Contour	GMR			IEEZ		
	Eastern (EE)	Northern (NE)	Western (WE)	Eastern (EE)	Northern (NE)	Western (WE)
Ecuadorian fleet 50%	57.7	0	30.3	0	39.6	22.4
Ecuadorian fleet 95%	95.0	90.5	100	59.9	99.7	94.8
Foreign fleet 50%	3.6	0	50.4	7	5.1	53.9
Foreign fleet 95%	59.5	67.2	98.7	61.1	89.6	93.1

**Table 4:** Percent of overlap of core effort areas (50% kernels) and spatial extent of fishing activities (95% kernels) by gear types.

Contour	Ecuadorian fleets						Foreign fleets					
	GMR			IEEZ			GMR			IEEZ		
	Eastern (EE)	Northern (NE)	Western (WE)	Eastern (EE)	Northern (NE)	Western (WE)	Eastern (EE)	Northern (NE)	Western (WE)	Eastern (EE)	Northern (NE)	Western (WE)
Drifting longlines 50%	0	0	0	0	30.1	0	18.3	0	54.3	3.8	36.7	40.2
Drifting longlines 95%	0	0	0	0.9	83.1	58.9	100	86.6	100	20.9	82.2	100
Unknown gears 50%	13.2	0	1.2	0	0	0	19.8	13.4	15.1	0	0	0
Unknown gears 95%	56.6	23.5	24.1	0	0	0	67.6	90.9	86.1	0.2	1.6	1.4
Pole and line 50%	12.4	0	0	0	0	0	32.7	0	62	0.4	0	7.6
Pole and line 95%	61.3	21.8	25.1	0	0	0	100	37	97.9	12.4	6	49.9
Tuna purse-seines 50%	0	8.8	25.6	10.9	25.7	52.4	3.2	0	48	6.8	5	53.6
Tuna purse-seines 95%	19.6	0	82.5	85.3	84	99.3	45.5	60.3	95.4	61.5	87.6	93.1

**Fig 6:**
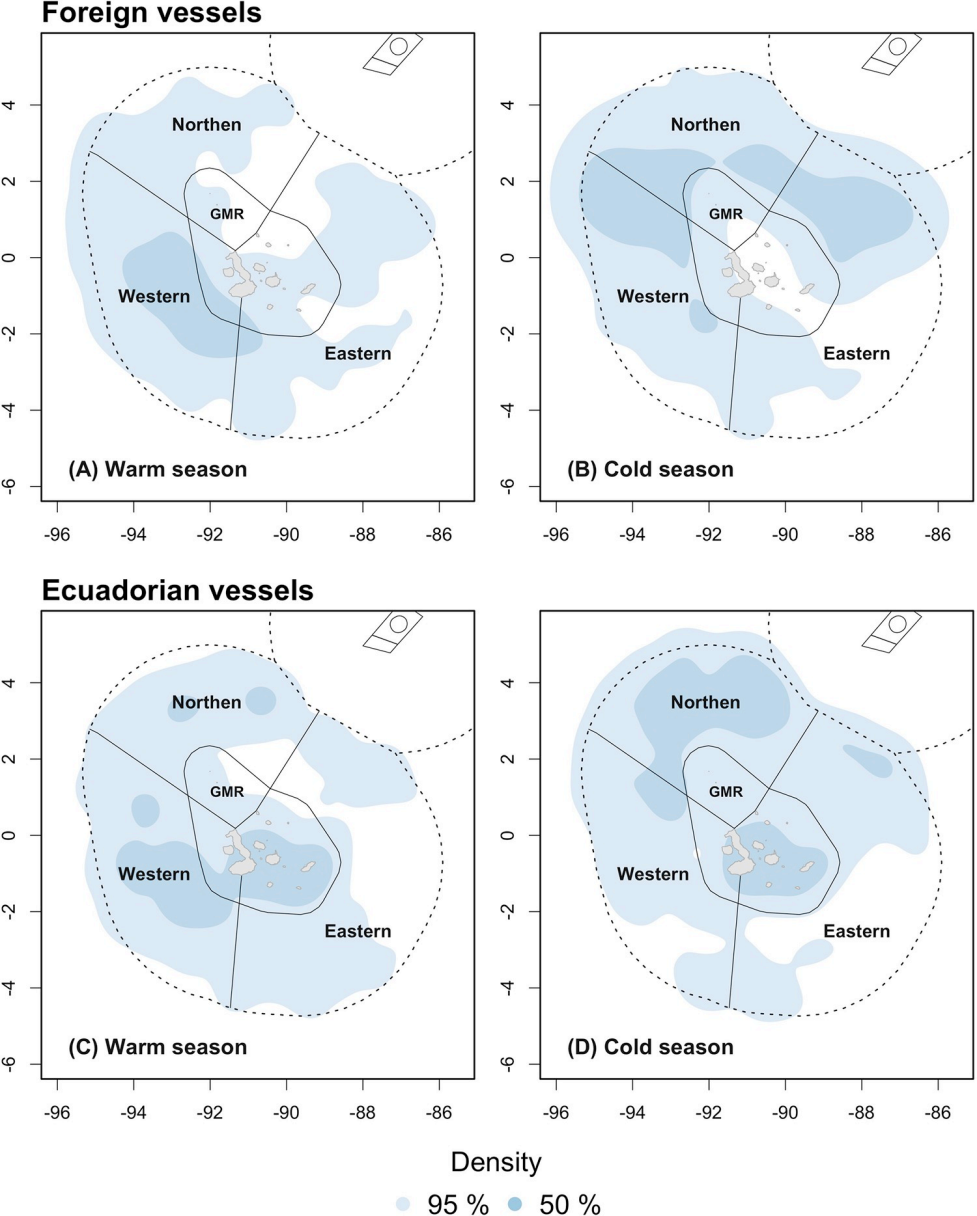
Spatial distribution of fishing effort by seasons. (A) Warm season and (B) cold season with fishing data for all foreign vessels. (C) Warm season and (D) cold season only with fishing data of the Ecuadorian vessels. Data from ‘Unknown flags’ was not used in these plots.

Drifting longlines were used in the IEEZ’s northern and western ecoregions by foreign Ecuadorian vessels, yet the latter maximum overlap reached 83% with the northern ecoregion (Table 4). Foreign fleets reported two core fishing grounds while the Ecuadorian fleet only one in the northern ecoregion. Fishing via other purse-seines, set gillnets and set longlines were carried out only by Ecuadorian vessels and within the GMR (S3 Fig), while squid jiggers were exclusively flagged to China and were used only in the southern border of the IEEZ. The foreign fleets concentrated their core fishing activities in the western ecoregion during the warm season, overlapping with a 31.6% of the IEEZ and 36% of the GMR portion of this ecoregion. Core fishing grounds covered broader areas during the cold season, overlapping with the northern (28.8%) and western (29.6%) IEEZs (Fig 6A and 6B, Table 5). The core fishing area of the Ecuadorian fleet in the IEEZ was mostly allocated in the western during the warm season (28.9% overlap), and in the northern during the cold season (55.3% overlap) (Fig 6C and 6D). This fleet also showed an important activity within the GMR during both seasons.

**Table 5:** Percent of overlap of core effort areas (50% kernels) and spatial extent of fishing activities (95% kernels) by seasons.

Contour	Ecuadorian fleets						Foreign fleets					
	GMR			IEEZ			GMR			IEEZ		
	Eastern (EE)	Northern (NE)	Western (WE)	Eastern (EE)	Northern (NE)	Western (WE)	Eastern (EE)	Northern (NE)	Western (WE)	Eastern (EE)	Northern (NE)	Western (WE)
Warm season 50%	55.4	0	38.7	0	6.2	28.9	2.5	0	36.6	1.5	0	31.6
Warm season 95%	93.7	75.5	100	60.1	83.1	94.8	93.7	75.5	100	60.1	83.1	94.8
Cold season 50%	49.3	1.0	18	2.3	55.3	6.9	0	9.6	3.0	20	28.8	29.6
Cold season 95%	100	100	100	53	100	84.1	54.5	83.5	97.8	60.5	97.5	88.7

## Discussion

Understanding the spatial distribution of fishing effort in a nation’s EEZ is useful for policy reformulation, particularly regarding access agreements that allow foreign fishing in national waters [51]. Our study provides detailed information on the main areas and fishing gears used by foreign and national fleets, which can contribute to improved spatial management of fisheries in the IEEZ and the GMR, particularly the less reported fisheries. The waters around the MPA were mostly targeted by tuna purse-seiners and drifting longlines, but purse-seiners fished much further inside the IEEZ than longliners (average distance of 159 and 107 km, respectively). The Ecuadorian fleet concentrated its fishing effort mainly in the northern ecoregion of the IEEZ and within the GMR, the latter likely due to the fishing activities of local Galápagos artisanal boats. Foreign fleets fished mostly near the GMR’s western boundary during the warm season, with a subtle shift observed towards its opposite boundary in the eastern ecoregion during the cold season. The implications of these results are discussed based on fishing effort by ecoregion, flags and areas, and the limitations of the used AIS data.

### Fishing effort by ecoregion

Our results highlighted that the eastern ecoregion exhibited the highest amount of fishing hours, with vessels operating more evenly distributed within the IEEZ yet clustered within the GMR. Differently, the western and northern ecoregions exhibited constant clustering in two areas: purse-seines preferring the western ecoregion near the border next to Isabela and Fernandina Islands, and drifting longlines the northern ecoregion in the area north to Darwin Island. Hu, Harrison [52] reported these two fishing fleets target the same tuna species (*T*. *albacares*, *T*. *obesus* and *K*. *pelamis*), yet there are differences in the habitat preferences of younger tunas (e.g. limited O_2_ concentration, shallow mixed density layer, and high productivity conditions) targeted by purse-seiners compared to those of older tunas (e.g. wide ranging O_2_ concentration, deeper mixed density layer, and low productivity conditions) generally targeted by drifting longlines. The GMR lies in the confluence of several surface and underwater marine currents whose interactions boost the overall biological productivity around the islands, particularly in the west of the reserve where the upwelling of nutrient-rich cold waters occurs [53]. These conditions create an area of relatively stable species-habitat relationship that favors the overall diversity of the area [54], which is taken advantage by foreign and Ecuadorean purse-seine fleets targeting tuna, billfish and swordfish [21, 22]. Notwithstanding, the Galápagos upwelling is known to expand towards the west during cold conditions but to concentrate near the west coast of Isabela and Fernandina Islands during the warm season or El Niño events [53, 55]. Since juvenile and subadult bigeye (*T*. *obesus*) and skipjack tunas (*K*. *pelamis*) targeted by purse-seiners are reported to prefer waters of high primary productivity [52], the strength and extent of the Galápagos upwelling is likely the underlining reason on the seasonal shift in fishing effort allocation by fleets targeting those species.

The northern ecoregion represents a unique "hotspot" for sharks [56], particularly over the seamounts found along the Cocos Ridge [57]. While previous analysis of AIS data did not depict this ecoregion as of high fishing interest [34], Martínez-Ortiz, Aires-da-Silva [30] denoted the area is an important fishing ground for the pelagic thresher shark (*A*. *pelagicus*) and blue marlin (*M*. *nigricans*). This is also supported by Cambra, Lara-Lizardi [57], who reported the presence of the scalloped hammerhead shark (*Sphyrna lewini*) and pelagic thresher shark aggregating over seamounts found along the Cocos Ridge. The high abundance of sharks over this region could be drawing the interest of the Ecuadorian and foreign drifting longline fleets during the cold season to fish on these species, as reported by Martínez-Ortiz, Aires-da-Silva [30]. Based on these findings, it is suggested the observed fishing patterns around the GMR from AIS data are tied to the seasonality of the oceanographic setting linked to the resource been targeted. This research did not use environmental data (e.g., biological productivity, sea surface temperature, currents), therefore we recommend additional studies to deepen the understanding of the fishing effort allocation in relation to these variables around the GMR by pairing AIS data with other sources of fishing effort and catch composition information, particularly to assess threatened and protected species. This type of information can also be used to make predictions of fishing activities based on environmental data to facilitate the monitoring and surveillance by enforcement authorities.

### Fishing by foreign flags

The observed fishing patterns by flag and gear type reported by our study align with previous work using AIS data (2012–2016) that examined fishing activity in Ecuadorean waters, yet found Colombia as the leading foreign flag operating within the IEEZ [34]. These differences may respond to the use of a bigger dataset processed with refined algorithms into our analysis, an increased number of fishing permits allocated to other countries (such as Panama), and, in the case of China, the steady increase in operations around Ecuador’s IEEZ since 2016 [58]. Our study also found the IEEZ and the GMR were fished by 13 foreign flags, of which seven do not belong to the coastal states of the Eastern Tropical Pacific Ocean. During 2012–2018, AIS data revealed that Eastern Tropical Pacific fishing effort was dominated mostly by Chinese and Taiwanese flag squid jiggers (64%), followed by tuna purse-seiners (17%) and drifting longlines (15%), both flagged to a wider range of countries [59]. Similar results have been reported in other EEZs, where distant-water fleets dominate fishing in these areas and often approach protected areas [60, 61]. The presence of distant-water fleets in the IEEZ may be due to Ecuador’s fishing agreements and permits with these countries. The United Nations Convention on the Law of the Sea (UNCLOS) gives nations exclusive rights to their open waters EEZ, meaning foreign vessel can operate within these under specific agreements through their State Departments [62]. During this research, a list of foreign vessels approved to fish in the IEEZ under this framework, and for what fisheries and time periods, could not be accessed. Care is thus advised while assessing this data to infer into potential illegal practices, since it is impossible to filter out the names and registry of the foreign vessels legally operating within Ecuador. This caution is also relevant to the observed fishing pressure from Chinese and Portuguese vessels in the limits of the IEEZ. While studies have reported Chinese vessels could be involved in illegal entries within the IEEZ [58, 63, 64], our method of analysis gridded the spatial information and used the centroid of the calculated grids to estimate fishing activity per cells. This could produce some records to be depicted as within the IEEZ, particularly for Portugal since it only recorded one fishing record with 2.4 fishing hours. A further in-depth analysis is highly recommended to (1) differentiate the foreign vessels with and without fishing permits, and (2) to assess the detailed track of each vessel without permits for understanding their patterns and level of incursion within the IEEZ. This complementary analysis and the subsequent adoption of cost-effective methods to tackle illegal fishing (e.g., increase monitoring patrols and radars in areas where fishing effort is higher) could help Ecuador regain confidence from the European Commission, who categorized Ecuador as a fully non-cooperative country in the fight against IUU fishing with a yellow card in 2019 [65].

### Fishing effort within GMR

A different scenario is observed for foreign vessels detected by Global Fishing Watch algorithms fishing within the GMR. For this MPA, only local (to Galápagos) artisanal fishers are allowed to fish and under specific gear type and fishing power characteristics [66]. Yet, since the creation of the GMR in 1998, there have been multiple of seizures of boats from Ecuador (mainland), Costa Rica, Colombia and Japan illegally operating within the marine reserve [20, 67–70]. Testimonies from Galápagos fishermen corroborate that unauthorized fishing by industrial vessels is recurrent within 40 nautical miles of this MPA [71]. Our study flag suspicious fishing activities from Colombia, Japan, Nicaragua, Panama, USA, Venezuela, Vanuatu, and ‘Unknown flags’ within the GMR. It is noteworthy that most of these records are in the southcentral region at an entry distance of nearly 100 km in the reserve. Out of these, Vanuatu and Japan are the only ones with a few fishing events registered close to port areas of the GMR (1 and 2 events, respectively), and not for the IEEZ. Since it is unlikely these vessels were fishing right in front of the main navy and park ranger offices, a potential misidentification by the algorithm could be the reason why those vessels were flagged as fishing.

Records from other vessels of known foreign flags represent a different scenario since most of them were registered operating near the western border or around islands but far from port areas. The GMR waters host important nursery and aggregation areas for commercial fish and protected shark species [72], making both the IEEZ and the GMR susceptible to infringements on fishery resources by national and foreign industrial fleets [69]. There is a possibility that vessels were found only in innocent passage, although the density of vessels transiting around the waters of the GMR is not as high as in other areas of the Eastern Tropical Pacific Ocean [73]. Either by fishing or transiting, the observed entries by our study represent suspicious activities within the reserve. A detailed analysis of the full AIS track data coupled with declared entry details to port authorities is highly recommended to detect common vessel behaviors that could lead to illegal fishing events within the reserve boundaries. This information could aid managers and authorities in developing tailored management and cost-effective surveillance plans for foreign and Ecuadorian industrial fishing boats entering the reserve under alleged health, mechanical or other emergencies [74].

### Local artisanal fleet

The concentration of fishing activities within the GMR observed by ‘Unknown flags’, or by Ecuadorean vessels using gears including other purse-seines, set gillnets, set longlines and unknown fishing gears, are attributable to the local (to Galápagos) artisanal vessels that are legally operating within the reserve. The spatial distribution of fishing effort of Galápagos artisanal boats is influenced by several geographical (e.g., latitude, longitude, homeport), oceanographic (e.g., El Niño-Southern Oscillation), social and economic (e.g., target species, vessel type, revenues) variables [75]. Fishing grounds of these vessels are found all over the archipelago, but the most frequently fished sites are close to the fishing ports [24, 75]. The Galápagos fleet is required by law to use AIS, even in the case of small fiberglass boats, and are authorized to fish with trolling, deep sea and mid-water hook and lines, coastal gillnets, and Hawaiian slings [23]. Under these conditions, it is possible the assigned fishing gears and unknown flags recorded within the GMR do not represent potential illegal fishing events but errors by the algorithm when assigning a fishing gear [12], particularly since the used classification does not represent all the possible fishing gears used within the GMR [76]. To avoid any confusion, records from unknown flags were removed from data analysis and were not displayed in the results section. However, the presence of a high number of ‘Unknown flag’ vessels detections (> 2,400) shows the existence of large gaps in vessels monitoring, which may represent a risk to the GMR because these vessels have often associated with IUU fishing practices [77]. Therefore, it is necessary to assess the extent of ‘Unknown flag’ fishing activities by employing strategies such as detailed monitoring of their tracks and port arrivals.

### Limitations of AIS data

Our study demonstrates the value of AIS data to analyze spatial and seasonal patterns of global fisheries in and around an MPA. Nevertheless, since this data does not provide information on fishing hours and the fishing gear used in itself [7], machine learning algorithms calculate a probability that a vessel is fishing based on the type of gear and its movements [10, 78, 79]. The identification of vessels and their fishing activities is limited by the fact that not all fishing vessels use this system [80], the ones that use it may present incorrect or incomplete identity data in the official vessel records [12], or because they choose to turn off the AIS to cover potentially illegal operations [81]. Another important limitation is that the algorithm cannot currently properly identify vessels that use multiple fishing gears or differentiate between their fishing activities [12].

The resulting gaps in AIS data are a substantial hurdle, and their use may be inappropriate for estimates of fishing intensity. To tackle this, Global Fishing Watch rates all designations of fishing effort or fishing activity as “apparent” and suggests considering the information as an estimate [82]. Catch reconstructions have shown that the Global Fishing Watch algorithm can provide reasonable estimates of fishing effort under optimal conditions (e.g., proper use and reception of AIS, the existence of vessel records with the type of fishing gear) [12], especially for areas where no or few prior data exist. Each year the coverage of global fishing activity is better due to the increase in the number of vessels that use AIS, the launch of more AIS-capable satellites [12], the latter approaching 100% global coverage, and the number of nations that publicly share their VMS data supported by Global Fishing Watch. Currently, ten countries including Ecuador, share their VMS data to this platform, but this information cannot be downloaded because it remains the property of the respective national authorities [46]. Previous research has used AIS data to map the fishing effort of fishing fleets [10, 44], assess fisheries management and marine planning [8, 9] and even analyze the overlap of fishing effort with threatened or endangered species [83, 84]. These studies have shown that AIS data, especially when combined with additional and complementary information such as VMS, can be a powerful tool for evaluating near real-time fishing activities as well as to support maritime spatial planning [11, 85].

## Conclusion

In this work we analyzed AIS-based fishing effort data over space and time (seasons) to characterize patterns of fishing activity and key players by flag state in commercial fisheries. This data can provide a significant step forward in achieving sustainable fisheries management and improving offshore maritime planning, because it provides data on less studied areas, gear types and flags in the IEEZ and GMR. However, this information and its interpretation should be used with caution due to the nature and limitations of the AIS data. The identification of apparent fishing activities by 13 foreign fleets in the IEEZ and within GMR further support the relevance of these waters for commercial fishing. Given this situation, it is important to adjust fishing permits with nations and reinforce law enforcement strategies in the southcentral region, where most of these suspicious fishing activities were recorded. Our study also revealed the predominance of vessels fishing with tuna purse-seines and drifting longlines in the northern and western ecoregions, probably because the oceanographic conditions create a highly productive area that favors the presence of species such as tuna, billfish, swordfish and sharks. Yet, we recommended to deepen the understanding of the fishing effort allocation in relation to the environmental data around the GMR.

## Supporting information

S1 Fig Locations of the AIS detections by flag during the study period. Numbers below each country name refer to the number of detections registered in only the IEEZ and the GMR. The purple polygon represents the northern ecoregion; the green, the eastern ecoregion, and the light blue, the western ecoregion.
(TIF)

S2 Fig Graphical representation of the calculation of method to calculate the entry distance for each AIS detection. (TIF)

S3 Fig Spatial distribution of fishing effort by fishing gear used only by Ecuadorian vessels. Data from ‘Unknown flags’ was not used in these plots.
(TIF)
