# Supplementary figures and images for: Fishing effort dynamics around the Galápagos Marine Reserve as depicted by AIS data
Source: PLoS One. 2024 Apr 3;19(4):e0282374. doi: 10.1371/journal.pone.0282374 (PMC10990170; doi:10.1371/journal.pone.0282374)

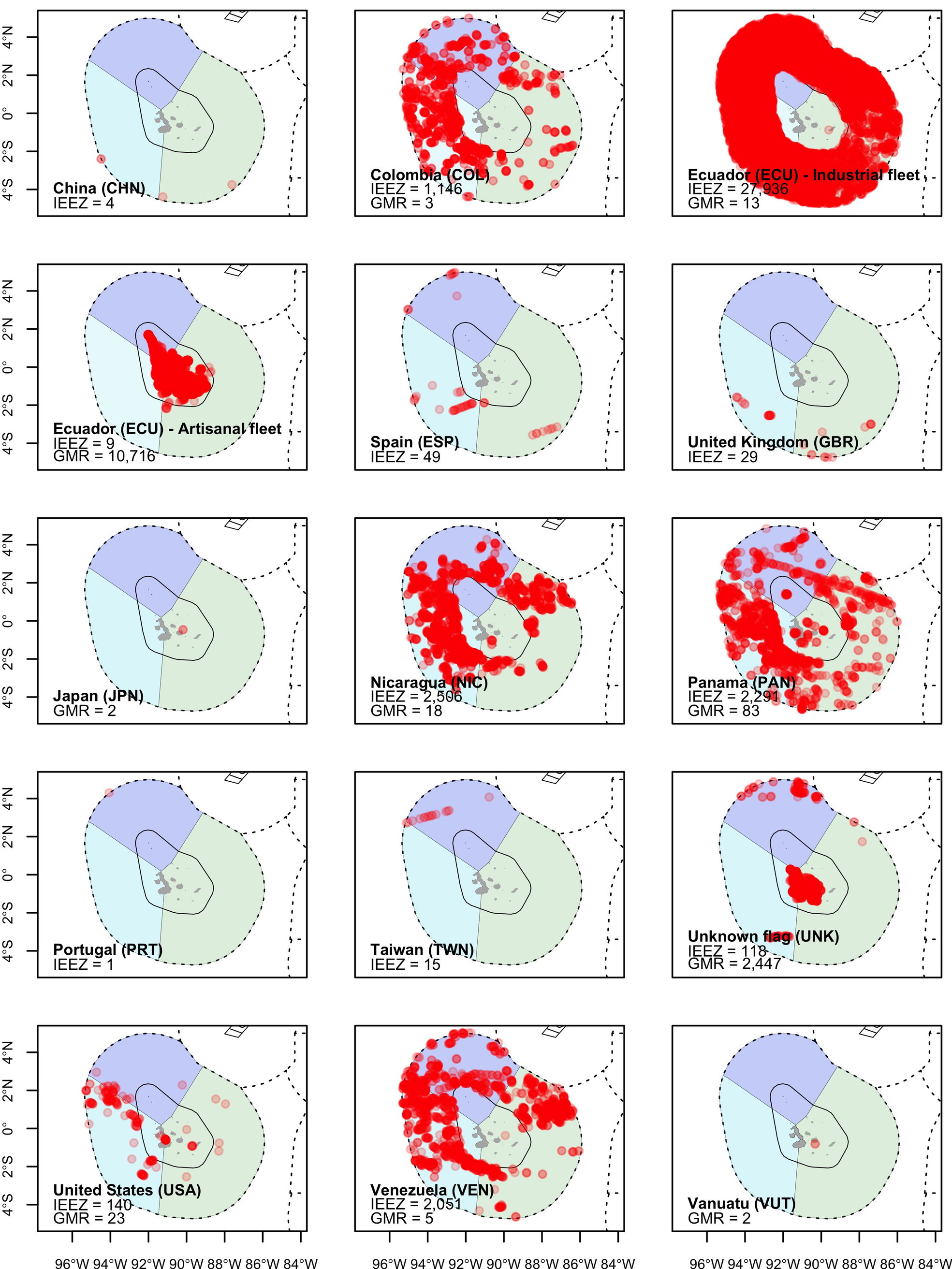

Supplement: S1 Fig — Numbers below each country name refer to the number of detections registered in only the IEEZ and the GMR. The purple polygon represents the northern ecoregion; the green, the eastern ecoregion, and the light blue, the western ecoregion. (TIF) [file pone.0282374.s001.tif]

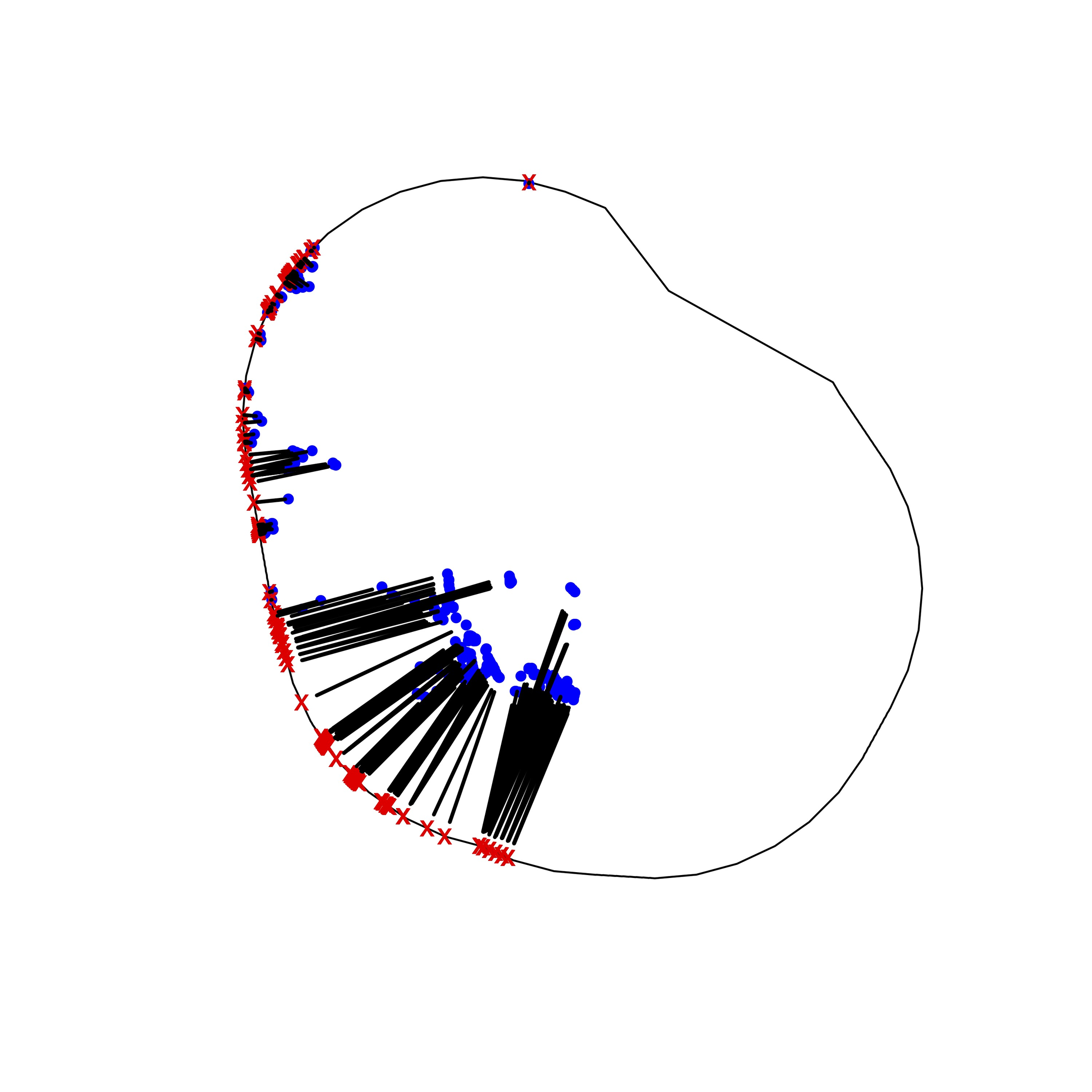

Supplement: S2 Fig — (TIF) [file pone.0282374.s002.tif]

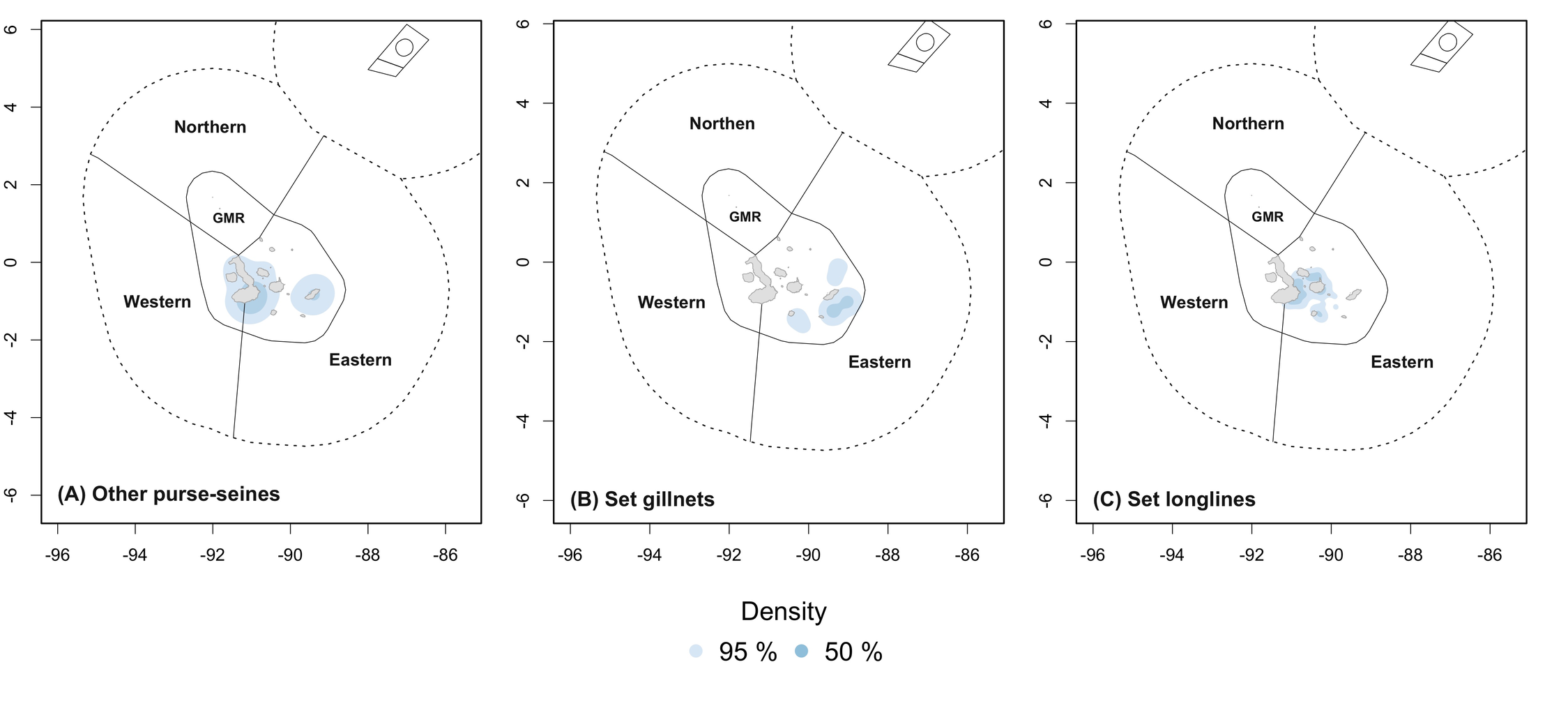

Supplement: S3 Fig — Data from ‘Unknown flags’ was not used in these plots. (TIF) [file pone.0282374.s003.tif]
